# Mentor mother support for mothers experiencing intimate partner violence in family practice: A qualitative study of three different perspectives on the facilitators and barriers of implementation

**DOI:** 10.1080/13814788.2016.1267724

**Published:** 2017-01-18

**Authors:** Maartje J.W. Loeffen, Jasper Daemen, Fred P.J.F. Wester, Miranda G.H. Laurant, Sylvie H. Lo Fo Wong, Antoine L.M. Lagro-Janssen

**Affiliations:** ^a^ Department of Primary and Community Care, Gender & Women’s Health, Radboud University Medical CenterNijmegenthe Netherlands; ^b^ Närhälsan Hjo Vårdcentral HjoSweden; ^c^ Faculty Social Sciences, Section Methods and Techniques, Radboud University NijmegenNijmegenthe Netherlands; ^d^ Scientific Institute for Quality of Healthcare, Radboud University Medical CenterNijmegenthe Netherlands

**Keywords:** General practice, treatment/intervention research, qualitative designs and methods, paraprofessional and mentor mother

## Abstract

**Background:** Intimate partner violence (IPV) is highly prevalent and associated with physical and mental health problems. Mentor mother support is a low threshold intervention in family practice consisting of support by non-professionals trained to support mothers experiencing IPV. A mentor mother support study showed reduced exposure to IPV and decreased symptoms of depression.

**Objectives:** Identify factors determining implementation success of mentor mother support in family practice.

**Methods:** Individual interviews were conducted with 12 family physicians, 16 abused mothers and three mentor mothers. Four mentor mothers participated in a focus group. Qualitative content analysis was used to analyse the data.

**Results:** The identification and discussion of abuse is hindered by family physicians’ attitudes because they considered mothers experiencing IPV as a difficult target group with a responsibility of their own to break out of their violent situation. Some family physicians doubted the partner’s violence because he was known as a patient as well. Acceptance of mentor mother support is related to the readiness for change of mothers experiencing IPV. Mentor mothers facilitate acceptance and completion of their support by connecting as a friend who is equal and less threatening than professionals.

**Conclusion:** To improve successful implementation of mentor mother support in primary care, we should focus on family physicians’ attitudes towards IPV. To change these attitudes, we recommend continuous training of family physicians. By being paraprofessional friends, mentor mothers offer low threshold support that is complementary to professional support and should be embedded more widely in primary care.

KEY MESSAGES

- We need to focus on family physicians’ attitudes towards IPV to improve the support for mothers experiencing IPV.
- As ‘paraprofessional friends,’ mentor mothers offer low-threshold support that is complementary to professional support.

## Introduction

Intimate partner violence (IPV) is a highly prevalent problem worldwide.[1] It is defined as physical, sexual, and/or emotional abuse by a partner or ex-partner and assumes power inequality between partners.[2]

IPV has many negative consequences for female victims of IPV, with increased levels of physical complaints such as injury and gastrointestinal symptoms, as well as mental disorders such as depression and posttraumatic stress.[3] Because of these negative health consequences, female victims of IPV visit their family physicians almost twice as often as women who never experienced IPV.[4] Some waiting room surveys in family practice record a lifetime prevalence of IPV between 30 and 45 per cent.[5–10] The family physician, therefore, is in a unique position to recognize and ask about abuse and intervene and offer help to this vulnerable group of women. However, family physicians often do not recognize the hidden symptoms of IPV and female victims of IPV find it very hard to disclose their problems.[11–13] When the family physician identifies IPV, it is important to offer an effective intervention that will be accepted by victims to diminish the harmful effects of IPV.[14]

An effective mentor mother support programme from Melbourne (MOSAIC) has been adapted to the Dutch situation in Rotterdam and Nijmegen. This programme—mentor mothers for support and advice (MeMoSA)—offers support during 16 weeks, with weekly visits by a mentor mother to support, empower, and educate mothers experiencing IPV.[14] Mentor mothers are paraprofessionals who received 10 days of training to learn how to support mothers experiencing IPV providing support at home or any other place, such as the family practice, where the mothers felt safe and comfortable. The mentor mothers were supported and supervised by a mentor social worker (Nijmegen) with training experience. Family physicians who participated in the MeMoSA study received a three hour-training program to improve recognition and discussion of IPV, and could refer mothers experiencing IPV to a mentor mother.

The study in Rotterdam showed positive results similar to those of MOSAIC favouring mentor mother support.[15] The main findings were: decreased exposure to IPV; reduced symptoms of depression; and increased social support, participation in society, and acceptance of mental healthcare for mother and child, making mentor mother support a very promising intervention.[15,16] The MeMoSA study in Nijmegen engaged in a process evaluation, aiming to identify factors from the perspective of the family physician, the mother who experienced IPV and the mentor mother, determining implementation success. This information served as the basis for recommendations to optimize the effects of intervention through mentor mother support in family practice.

## Methods

### Study design

We designed an observational implementation study that took place in the Netherlands from January 2011 to June 2012. The details of the study and the intervention have been described in the study protocol published by Loeffen et al.[14]

During the study, 35 eligible mothers who experienced IPV were identified by 28 participating family physicians. Eight women were offered mentor mother support but did not accept support because they preferred another kind of help (*n* = 2), or because they were not ready to disclose the abuse and accept any help (*n* = 6). Out of the 27 abused mothers who started, nine left the programme prematurely for the following reasons: (1) time restraints (*n* = 2); (2) other (professional) help (*n* = 2); (3) the mentor mother support did not meet her expectations (*n* = 1); and (4) no clear reason (*n* = 4).

### Research population and data collection

We conducted semi-structured interviews with family physicians, mothers who experienced IPV and mentor mothers. The interview and focus group guide (Supplementary material, available online) was developed based on literature and discussed in an expert panel (SLFW, FW, MLa, AL). In the mentor mother process, we distinguished four subsequent phases that are essential for successful implementation in family practice. In the identification phase, first, the family physician had to identify and discuss IPV. In the referral phase, second, the family physician had to refer a mother experiencing IPV to a mentor mother. In the acceptance phase, third, the mother experiencing IPV had to accept the mentor mother support that was offered by the family physician. In the last completion phase, fourth, mothers experiencing IPV had to be supported during 16 weeks and had not prematurely terminated the support that had to be offered. The interview and focus group guide focused on the facilitators and barriers at each subsequent phase as described above. Questions for the family physicians mainly pertained to the identification and referral phase and questions for the mothers experiencing IPV and the mentor mothers mainly related to the acceptance and completion phase. All interviews were fully recorded, transcribed verbatim and processed using ATLAS.ti 7.

#### Family physicians

Originally, we had planned to conduct focus group discussions for participating family physicians,[14] but due to difficulties in planning focus group sessions for family physicians, we changed to individual interviews. We selected 12 family physicians by purposive sampling of referring versus non-referring, male versus female, and rural versus urban family physicians to pursue maximum diversity. Ten family physicians were interviewed by a research assistant (VP) and two were interviewed by a researcher (MLo).

#### Mothers experiencing IPV

All 18 mothers who finished the programme and signed an informed consent form to be interviewed were invited for an interview six months after the start. The interviewer contacted them by phone and made an appointment for the interview to take place in a setting where they felt safe and comfortable. Two trained research assistants (MS, HH) performed the interviews. We succeeded in talking to 16 mothers who experienced IPV and their characteristics are shown in Table 1. Two mothers could not be reached after several attempts to contact them.

**Table 1.** Characteristics of mothers experiencing intimate partner violence (*n* = 16).

Age category (years)	*n* (%)
18–25	3 (19)
26–35	3 (19)
36–45	5 (31)
46–55	5 (31)
Country of origin	
Netherlands	11 (69)
Turkey	1 (6)
Antilles	2 (13)
Morocco	2 (13)
Number of children	
1	7 (44)
2	4 (25)
3	4 (25)
4	1 (6)
Living situation	
With partner and child(ren)	8 (50)
With children	6 (38)
Other	2 (13)
Education level^a^	
Low	5 (31)
Middle	7 (44)
High	4 (25)

^a^ Education level, low: no school/primary school/lower vocational education, middle: middle vocational education/higher general secondary education/pre-university secondary education, high: higher vocational education/university.

#### Mentor mothers

Of the eight mentor mothers who were employed and trained for the MeMoSA Nijmegen study, four completed their job during the project and participated in a focus group, facilitated by a research assistant (VP). Four mentor mothers resigned from their jobs before the end of the project because they were unable to combine their job as a mentor mother with other activities. Three of them were interviewed individually (MLo). One mentor mother who withdrew prematurely could not be reached.

### Data analysis

Two researchers (MLo, JD) analysed the qualitative data from the interviews and focus group, using content analysis consisting of open, axial and selective coding steps as described by Corbin and Strauss.[17] First, they started by reading four interviews with the family physicians, four interviews with women experiencing IPV, one interview with the mentor mother, and the focus group. The researchers (MLo, JD) independently coded these interviews and this focus group. In the next step, they compared and discussed codes with each other.[17] If differences in coding a segment occurred; the two researchers reread and discussed the text until consensus was reached, with the help of a third independent researcher (AL), if needed. The axial coding resulted in a final code list that was used to code all other interviews. Additional codes that emerged from these interviews were also applied to the previously coded transcripts. In the end, all transcripts were analysed with the same codebook. Themes were constructed by grouping all codes into categories by the two researchers (MLo and JD) who also coded the interviews and focus group. These themes were discussed with the supervising committee (AL, SLFW, FW and MLa) and changed if needed. Finally, the interviews and focus group were selectively coded with these themes in mind.

In this study protocol, we described our plan to analyse the facilitators and barriers as these related to the individual, the social context, the organization, and society based on Grol et al.[14,18] During our content analysis, however, we found it was preferable to classify our results in line with the four subsequent phases as described above identification, referral, acceptance and completion.

## Results

In the analysis process, we distinguished the following four subsequent phases the mothers experiencing IPV were going through: the identification phase, the referral phase, the acceptance phase, and, finally, the completion phase.

**Figure 1.**
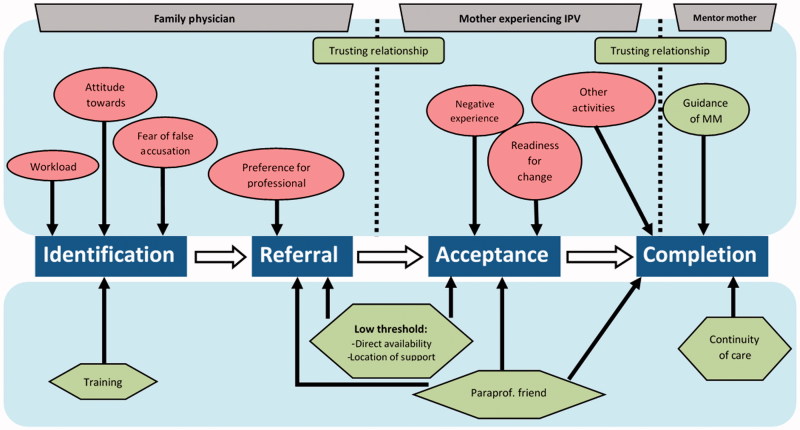
Facilitators and barriers of implementing mentor mother support.

In each phase, we found facilitators and barriers that influenced the implementation. Therefore, we have described our main themes into these four phases and have presented them schematically in Figure 1. The main results have been illustrated by means of quotes.

### Identification phase

The family physicians’ attitude towards IPV revealed the reluctance to ask about violence on their part because they regarded female victims of IPV as ‘difficult patients’.

[The interviewer asked the family physician why she decided to participate in the study]*… to improve patient care, and I thought it is something new; you have to try some things. However, I am sceptical about it because I know from experience that these people withdraw from all interventions and do not want anyone to meddle with them. It is a difficult target group*. (Family physician, female, age 59.)

Some family physicians doubted whether it was their task to discuss IPV proactively. They expected female victims of IPV to take their responsibility to break out of the violent situation, in contrast to situations with battered children, in which they felt more responsible for intervening.

*Sometimes I ask myself, honestly, does it all belong to the family physician’s task, and do we have to do it all, inquire and feel inadequate if she does not disclose the abuse … they can ask me for anything, but patients have to take some initiative as well*. (Family physician, female, age 59.)

Other family physicians mentioned that, although abuse is not permitted, if an interaction between the partners led to a violent situation, both contributed to the abuse and both, therefore, had a responsibility to end the violent situation. Some family physicians could not imagine that a specific partner was violent because they knew him as a patient as well.

*Sometimes it is unclear whether the man is the perpetrator, … it is difficult to assess where the problem is located. Yes, violence is not allowed, that much is clear, but I know some families where both of them are aggressive*. (Family physician, female, age 46.)

Family physicians who took a proactive attitude towards IPV identify the abuse more easily:

*Generally, I try to make another appointment, so it is not that is over yet. You have to push a little bit, … that is a little bit my style, I think that’s why I attract it, that I see it a lot, that’s just the way it works*. (Family physician, female, age 36.)

Furthermore, the family physicians expressed that fear of false accusation could lead to a serious breakdown of trust between the family physician and a female patient or her partner, who are often both patients of the same family physician.

*Of course, you have to discuss it if the one verbally or physically threatens the other and sometimes you have to raise the issue yourself because you see it happen. I think that it is the feeling that you are going to lose contact with either of the two patients*. (Family physician, male, age 53.)

The family physicians’ high workload was a hindering factor for identifying IPV. They mentioned that their time was too short to discuss delicate subjects such as IPV in particular. Abuse was considered a complex problem, involving vague and diverse complaints, making it hard to recognize.

*There is your time pressure; you do not always want to or have the time to, so you just focus on the complaint…. Sometimes your hands are full with the somatic part*. (Family physician, female, age 57.)

Finally, the family physicians suggested that more and continuous training was needed to improve their competences regarding identification and discussion of IPV.

### Referral phase

The family physicians valued the direct availability of the mentor mother, whereas there were often waiting lists for access to professional support.

All three groups of respondents also valued the fact that mentor mothers offered support at home. Family physicians and mentor mothers especially mentioned that they acquired a better understanding of the social context of the mother experiencing IPV.

*The mentor mother’s observations; What does the house look like? Is it clean? How does the mother interact with the children? These important things give a lot of information*. (Family physician, male, age 58.)

A barrier to referral was that some family physicians preferred professional help if they considered a victim’s problems, mostly psychiatric problems, too complex. Nevertheless, family physicians regarded mentor mother support as a low-threshold intervention because the mentor mothers function as ‘paraprofessional friends’ (see the phase of acceptance for more details) who are considered less threatening than professionals.

[Family physician describes the mentor mother as] *someone who stands beside rather than above the patient … more of an equal, not someone they have to look up to or for whom they have to keep up appearances*. (Family physician, female, age 59.)

### Acceptance phase

To accept mentor mother support, the mother experiencing IPV needed to be ready for change, which was a recurring theme during the interviews with all respondents. Female victims of IPV needed to be aware of the abuse, be open to accepting help, and take action to get help.

*I have the feeling that it is also a process of growth for people. It has to grow, and they have to become aware that it is not normal … that violence is not allowed*. (Family physician, male, age 53.)

*I was reluctant; I told nothing to anyone. It was a very big step to go to the doctor and to talk about it.* (Mother experiencing IPV, age 26.)

Acceptance of support by mothers experiencing IPV was made more difficult by their negative experiences, as well as by shame and guilt, leading to reduced help seeking behaviour and scepticism towards support, especially professional support.

*I also told my doctor: I do not want to go to a psychologist or psychiatrist, they give me the feeling I am talking to a wall*. (Mother experiencing IPV, age 30.)

A trusting relationship between the family physician and the mother experiencing IPV was a prerequisite for accepting support.

*I was a bit sceptical at first, but because I do trust my family physician a lot, I thought let us try it*. (Mother experiencing IPV, age 49.)

The most important facilitator for acceptance of mentor mother support was the fact that the mentor mother operated as a ‘paraprofessional friend.’ All parties appreciated the mentor mother as a non-judgmental attentive listener, with the empathy and engagement of a friend on an equal level with the mother experiencing IPV, while being an expert and keeping a professional distance.

*It was just a friend [mentor mother], but also with more expertise. I considered her more as a kind of family physician you go to for a chat, or in some cases as a friend who comes to visit you…. You could ask more advice from her than from a friend*. (Mother experiencing IPV, age 30.)

*You are not connected to an institute, in that way you are very free and very neutral … now I am the woman next door, the next moment you are more like the professional … freedom to be very open because there was only little distance, enough though, more than with a friend*. (Mentor mother, age 45.)

### Completion phase

For the support process to be completed, a trusting relationship between the mother who experienced abuse and the mentor mother was mentioned as being essential. Mentor mothers were themselves mothers, who had sometimes experienced IPV themselves, which made them experts by personal experience. The mothers experiencing IPV felt well understood which facilitated completion of support. Additionally, they valued the mentor mothers’ open and interested attitude and their friendliness and connectedness.

*We* [abused mother and mentor mother] *had a great click so to speak especially as humans and that was the most important…. It was an hour every week, just an hour where I could tell my story and for me it really was one hour, that made me feel better*. (Mother experiencing IPV, age 45.)

Care for children and demands of work, of both the women experiencing IPV and the mentor mothers, sometimes made it difficult to meet every week and appeared to be a barrier to continuity of care.

*At first, I thought leave me* [abused mother] *alone, it is not that way. However, she [mentor mother] was very persistent. She said: “You think it goes well now, but you just wait and see, I have to continue to make appointments with you.”* (Mother experiencing IPV, age 39.)

*I had someone who I had to call every Monday so that she would remember our appointment on Tuesday or Wednesday … by visiting her weekly you learn that, well sometimes things go well and the next time it is a big step backwards*. (Mentor mother, age 46.)

Guidance and support for mentor mothers during the mentor mother programme was also considered valuable for managing the frustrations and emotions that accompanied their work with mothers experiencing IPV. Although this was important, there was not always enough time to focus on these feelings.

*In our team meetings, we always had a very tight schedule and there was no time to talk about my feelings*. (Mentor mother, age 47.)

## Discussion

### Family physicians’ attitude

First, we found that identification and discussion of abuse is hindered by family physicians’ attitudes towards IPV, which is also described, as a barrier for identification of IPV in other studies.[19,20] Family physicians question whether proactively discussing IPV is their job and often expect mothers experiencing IPV to take personal responsibility for reporting IPV and asking for help. They often encounter a lot of resistance in their consultation with female victims of IPV and, therefore, consider them a difficult target group. Furthermore, they feel frustrated or powerless because they are unable to solve the problem of IPV. Other studies also describe these feelings of frustration, powerlessness and uncertainty because healthcare providers are unable to ‘fix’ this complex problem.[21–23] Some family physicians, moreover, doubt whether the perpetrator is to blame entirely and believe the victim contributes to the violence as well. In other studies ‘blaming the victim’ has also been described as an attitude hindering identification of IPV.[19,20,24,25] We believe that training is necessary for family physicians to become more confident by learning how to cope with the issues involved in IPV. As family physicians are also part and parcel of society and are influenced by its cultural norms, we also believe that it will not be sufficient to focus on the individual level alone, and that attitude in society needs to change as well.

### Women’s readiness for change

Second, women’s readiness for change is an important factor that hinders or facilitates identification of abuse and acceptance of mentor mother support. The readiness, or stages of change, are based on the transtheoretical model of health behaviour change and have been described for IPV.[26] This model distinguishes five different stages that require a different approach. At the stage of pre-contemplation, for instance, the mother experiencing IPV has to become aware of the abuse by validating her experiences and reinforcing the unacceptability of IPV, while, at the stage of action, interventions and strategies need to be evaluated.[27] Family physicians’ feelings of frustration and powerlessness may be assuaged once they understand experiences of female victims of IPV and know that IPV victims often return to an abusive partner. As women who are unaware of IPV or not ready to accept help will not accept support, family physicians need to consider their readiness for change in order for doctors to be able to offer help that suits the victims’ stage of change. Training of family physicians, therefore, should also focus on recognizing the readiness for change of a women experiencing IPV and teach them how to deal with it.

### Mentor mothers as paraprofessional friends

Third, mentor mothers play a unique role as paraprofessional friends in accepting and completing support. When professionals are involved, mothers who are victims of IPV often fear they will lose their children because professionals are mandated to report child abuse; when mentor mothers are involved they act as a friend, which makes them less threatening than professionals. Mentor mothers provide the moral support and understanding that is needed to empower female victims actively to change their abusive situation, especially at an early stage of change.[12] By empowering female victims of IPV their wish to preserve autonomy can be met.[28] The bond between mentor mothers and victims, furthermore, helps to sustain a proactive attitude that prevents premature termination of support. Finally, the mentor mother’s professional guidance will enable them to handle the emotional burden that accompanies working with women experiencing IPV.

### Strengths and limitations

This study offers new insights into the factors that facilitate or hinder implementation of mentor mother support in family practice and especially emphasize family physicians’ attitudes towards IPV as a barrier for identification and discussion of abuse. It demonstrates the possible value of mentor mother support in family practice.

One of the limitations of this study is that not all participating family physicians by purposive sampling were interviewed. We tried to select a variable group of family physicians by purposive sampling to overcome this limitation. After interviewing 12 family physicians, we reached data saturation. Besides, only mothers experiencing IPV who completed the mentor mother support programme were interviewed, while mothers who rejected or withdrew from the mentor mother support programme prematurely, might have provided more insight into implementation barriers.

### Implications for practice

First, we recommend more and continuous training of family physicians, which should focus on their attitudes and on recognizing the stages of change of female victims of IPV. Although we believe this will improve identification and discussion of abuse, we also argue for changes at the level of society, because family physicians will not be able to solve this complex problem alone.

Second, broader embedding of low-threshold support in primary care should be considered to increase acceptance of help.

The authors strongly recommend the further development of low-threshold interventions that are more easily available and less threatening than professional support.

## Conclusion

Identification and discussion of abuse by family physicians are hindered by their attitudes towards IPV. Mentor mothers can fulfil a unique and complementary role as paraprofessional friends at a level of equality but equipped with the professional expertise that is needed to offer appropriate support.

## Supplementary Material

Appendix_A

## References

[1] Garcia-Moreno C, Jansen HA, Ellsberg M, et al Prevalence of intimate partner violence: Findings from the WHO multi-country study on women’s health and domestic violence. Lancet. 2006;368:1260–1269. 17027732 10.1016/S0140-6736(06)69523-8

[2] Krug EG, Dahlberg LL, Mercy JA, et al World report on violence and health. Geneva: World Health Organization; 2002.

[3] Campbell J, Jones AS, Dienemann J, et al Intimate partner violence and physical health consequences. Arch Intern Med. 2002;162:1157–1163. 12020187 10.1001/archinte.162.10.1157

[4] Prosman GJ, Lo Fo Wong SH, Bulte E, et al Healthcare utilization by abused women: A case control study. Eur J Gen Pract. 2012;18:107–113. 22519713 10.3109/13814788.2012.675503

[5] Richardson J CJ, Petruckevitch A, Chung WS, et al Identifying domestic violence: Cross sectional survey of women attending general practice. Br Med J. 2002;324:274–280.11823360

[6] Bradley F, Smith M, Long J, et al Reported frequency of domestic violence: Cross sectional survey of women attending general practice. Br Med J. (Clinical research ed). 2002;324:271. 10.1136/bmj.324.7332.271 PMC65059 11823359

[7] Hegarty KL, Bush R. Prevalence and associations of partner abuse in women attending general practice: A cross-sectional survey. Aust and NZ J Public Health. 2002;26:437–442. 10.1111/j.1467-842x.2002.tb00344.x 12413288

[8] Lokhmatkina NV, Kuznetsova OY, Feder GS. Prevalence and associations of partner abuse in women attending Russian general practice. Fam Pract. 2010;27:625–631. 20591818 10.1093/fampra/cmq044

[9] Prosman GJ, Jansen SJ, Lo Fo Wong SH, et al Prevalence of intimate partner violence among migrant and native women attending general practice and the association between intimate partner violence and depression. Fam Pract. 2011;28:267–271. 21239469 10.1093/fampra/cmq117

[10] Elliott BA, Johnson MM. Domestic violence in a primary care setting. Patterns and prevalence. Arch Fam Med. 1995;4:113–119. 7842148 10.1001/archfami.4.2.113

[11] Ramsay J, Richardson J, Carter YH, et al Should health professionals screen women for domestic violence? Systematic review. Br Med J. (Clinical research ed). 2002;325:314. 10.1136/bmj.325.7359.314 PMC117773 12169509

[12] Prosman GJ, Lo Fo Wong SH, Lagro-Janssen AL. Why abused women do not seek professional help: A qualitative study. Scand J Caring Sci. 2014;28:3–11. 23350912 10.1111/scs.12025

[13] Hegarty KL, Taft AJ. Overcoming the barriers to disclosure and inquiry of partner abuse for women attending general practice. Aust and NZ J Public Health. 2001;25:433–437. 11688623

[14] Loeffen MJ, Lo Fo Wong SH, Wester FP, et al Implementing mentor mothers in family practice to support abused mothers: Study protocol. BMC Fam Pract. 2011;12:113. 22008270 10.1186/1471-2296-12-113 PMC3209440

[15] Prosman GJ, Lo Fo Wong SH, Lagro-Janssen AL. Support by trained mentor mothers for abused women: A promising intervention in primary care. Fam Pract. 2014;31:71–80. 24132592 10.1093/fampra/cmt058

[16] Prosman GJ, Lo Fo Wong SH, Romkens R, et al ‘I am stronger, I’m no longer afraid…’, an evaluation of a home-visiting mentor mother support programme for abused women in primary care. Scand J Caring Sci. 2013;28:724–731. 24251372 10.1111/scs.12102

[17] Strauss A, Corbin J. Basics of qualitative research; techniques and procedures for developing grounded theory. London: Sage Publications; 1998.

[18] Grol R, Wensing M. Implementatie: Effectieve verbetering van patiëntenzorg (Implementation: Effective improvement of patientcare). Maarssen: Elsevier gezondheidszorg; 2006.

[19] Sugg NK, Thompson RS, Thompson DC, et al Domestic violence and primary care. Attitudes, practices, and beliefs. Arch Fam Med. 1999;8:301–306. 10418535 10.1001/archfami.8.4.301

[20] Taft A, Broom DH, Legge D. General practitioner management of intimate partner abuse and the whole family: Qualitative study. Br Med J. 2004;328:618. 14766719 10.1136/bmj.38014.627535.0B PMC381135

[21] Lo Fo Wong SH, De Jonge A, Wester F, et al Discussing partner abuse: Does doctor’s gender really matter? Fam Pract. 2006;23:578–586. 16595542 10.1093/fampra/cml004

[22] Lo Fo Wong S, Wester F, Mol S, et al ‘I am not frustrated anymore.’ Family doctors’ evaluation of a comprehensive training on partner abuse. Patient Educ Couns. 2007;66:129–137. 17317076 10.1016/j.pec.2006.12.013

[23] Williston CJ, Lafreniere KD. ‘Holy cow, does that ever open up a can of worms’: health care providers’ experiences of inquiring about intimate partner violence. Health Care Women Int. 2013;34:814–831. 10.1080/07399332.2013.794460 23790127

[24] Easteal PW, Easteal S. Attitudes and practices of doctors toward spouse assault victims: An Australian study. Violence Vict. 1992;7:217–228. 1294237

[25] Garimella R, Plichta SB, Houseman C, et al Physician beliefs about victims of spouse abuse and about the physician role. J Women’s Health Gend Based Med. 2000;9:405–411. 10868613 10.1089/15246090050020727

[26] Reisenhofer S, Taft A. Women’s journey to safety—the transtheoretical model in clinical practice when working with women experiencing intimate partner violence: A scientific review and clinical guidance. Patient Educ Couns. 2013;93:536–548. 24007763 10.1016/j.pec.2013.08.004

[27] Feder GS, Hutson M, Ramsay J, et al Women exposed to intimate partner violence: Expectations and experiences when they encounter health care professionals: A meta-analysis of qualitative studies. Arch Intern Med. 2006;166:22–37. 16401807 10.1001/archinte.166.1.22

[28] Chang JC, Cluss PA, Ranieri L, et al Health care interventions for intimate partner violence: What women want. Womens Health Issues. 2005;15:21–30. 15661584 10.1016/j.whi.2004.08.007

