# Supplementary material for: Mentor mother support for mothers experiencing intimate partner violence in family practice: A qualitative study of three different perspectives on the facilitators and barriers of implementation
Source: Eur J Gen Pract. 2017 Jan 18;23(1):27–34. doi: 10.1080/13814788.2016.1267724 (PMC5774271; doi:10.1080/13814788.2016.1267724)
Supplement: Appendix_A [file igen_a_1267724_sm9488.doc]

**Appendix A**

Theme lists interviews family physicians, focus groups mentor mothers and interviews mothers experiencing IPV

Interviews family physicians

1. Experiences with the women experiencing IPV and mentor mothers
2. Identification and discussion of IPV: facilitators and barriers
3. Referral to a mentor mother: facilitators and barriers
4. Experiences with professional support
5. Mentor mother support compared to professional support
6. Reasons to accept or refuse mentor mother support

Focus groups / interviews mentor mothers

1. Experiences as a mentor mother: strengths and improvements
2. Objectives of mentor mother support: facilitators and barriers
3. Acceptance of mentor mother support: facilitators and barriers
4. Completion of mentor mother support: facilitators and barriers
5. Reason(s) to resign from their job as a mentor mother

Interviews mothers experiencing IPV

1. Reason(s) to accept mentor mother support
2. Other support offered? Why choosing mentor mother support?
3. Additional support during mentor mother support
4. Experiences with mentor mother support: what went well, what went wrong, what could be improved?
5. Reasons to stop or continue mentor mother support
